# Vaccine based on a ubiquitous cysteinyl protease and streptococcal pyrogenic exotoxin A protects against *Streptococcus pyogenes *sepsis and toxic shock

**DOI:** 10.1186/1476-8518-6-8

**Published:** 2008-10-31

**Authors:** Robert G Ulrich

**Affiliations:** 1 Laboratory of Molecular Immunology, Army Medical Research Institute of Infectious Diseases, 1425 Porter Street, Frederick, Maryland 21702, USA

## Abstract

**Background:**

The gram-positive bacterium *Streptococcus pyogenes *is a common pathogen of humans that causes invasive infections, toxic-shock syndrome, rheumatic fever, necrotizing fasciitis and other diseases. Detection of antibiotic resistance in clinical isolates has renewed interest in development of new vaccine approaches for control *S. pyogenes *sepsis. In the study presented, a novel protein vaccine was examined. The vaccine was based on a recombinant protein fusion between streptococcal pyrogenic exotoxin B (SpeB), a cysteinyl protease expressed by all clinical isolates, and streptococcal pyrogenic exotoxin A (SpeA), a superantigen produced by a large subset of isolates.

**Results:**

A novel protein was produced by mutating the catalytic site of SpeB and the receptor binding surface of SpeA in a fusion of the two polypeptides. Vaccination of HLA-DQ8 transgenic mice with the SpeA-SpeB fusion protein protected against a challenge with the wild-type SpeA that was lethal to naïve controls, and vaccinated mice were protected from an otherwise lethal *S. pyogenes *infection.

**Conclusion:**

These results suggest that the genetically attenuated SpeA-SpeB fusion protein may be useful for controlling *S. pyogenes *infections. Vaccination with the SpeA-SpeB fusion protein described in this study may potentially result in protective immunity against multiple isolates of *S. pyogenes *due to the extensive antibody cross-reactivity previously observed among all sequence variants of SpeB and the high frequency of SpeA-producing strains.

## Background

*Streptococcus pyogenes *is a perennial human pathogen, causing mild infections and life-threatening diseases including pharyngitis, impetigo, necrotizing fasciitis, streptococcal toxic shock syndrome and rheumatic heart disease. Antibiotic-resistant strains are increasing in global distribution [1,2], and a marked worldwide increase in the prevalence of serious invasive disease caused by *S. pyogenes *has occurred in the last two decades [3,4], perhaps due to the emergence and distribution of more virulent strains. Although the incident is low, the recorded overall mortality rate is 45% among streptococcal toxic shock-like syndrome cases [5].

There are currently no licensed vaccines available for protection against diseases caused by *S. pyogenes*. Ideally, a vaccine should incorporate antigens from a major virulence determinant or antigens that are ubiquitously expressed by disparate bacterial strains. Streptococcal pyrogenic exotoxin A (SpeA) and other secreted superantigen toxins are potential candidates for vaccines because these proteins are associated with many outbreaks of streptococcal toxic shock syndrome and are virulence factors for invasive infections. In addition, bacteremia is commonly associated with cases of streptococcal toxic shock [6]. The secreted polypeptide of SpeA (25,700 M_r_) is classified as a superantigen [7] that facilitates bacterial immune escape by targeting the primary recognition step in adaptive immunity. The cellular receptors for SpeA are human major histocompatibility complex (MHC) class II molecules, primarily HLA-DQ and HLA-DR proteins expressed on select cell lineages, and the antigen receptors of T cells (TCRs). The normal antigen-specific signal transduction of T cells is disengaged by SpeA, displacing contacts of MHC-bound antigenic peptides with antigen combining site elements of the TCR, and results in an elevated polyclonal activation of T cells. Toxic shock may ensue from pathological levels of tumor necrosis factor alpha (TNF-α) and other pro-inflammatory cytokines released in response to secreted superantigens [8,9].

Most, if not all, *S. pyogenes *M protein serotypes express an extracellular cysteine protease (streptopain) historically termed streptococcal pyrogenic exotoxin B (SpeB), though not homologous in structure or function to SpeA or any other superantigen. The secreted protease SpeB is also a bacterial surface molecule with binding activity to laminin and other glycoproteins [10], making it a potential target of neutralizing antibodies. Further, SpeB is an important colonization and pathogenicity factor [11], reported to modify several host substrates. For example, the interleukin 1β precursor is cleaved by SpeB to produce active interleukin 1β [12], and the extracellular matrix proteins fibronectin and vitronectin are also cleaved [13], thus modulating entry of *S. pyogenes *into host cells [14]. Although multiple alleles exist, polyclonal antisera generated against SpeB from any strain react with SpeB from all *S. pyogenes *M1 serotypes examined [15]. Further, antibodies against SpeB are detected in patients with invasive *S. pyogenes *infections of either streptococcal toxic shock syndrome and/or necrotizing fasciitis [16]. The ubiquitous expression of SpeB by *S. pyogenes *strains and the conserved nature of the antigenic determinants recognized by antibodies are noteworthy features, thus fulfilling major criteria for a potential vaccine. Collectively, these observations prompted the presently described development of a fusion protein comprised of SpeA and SpeB that was used as a vaccine in experimental models of streptococcal toxic shock and sepsis.

## Methods

### Recombinant streptococcal proteins

Genes encoding SpeA (M19350) and SpeB (M86905) were cloned from a clinical laryngitis isolate of *Streptococcus pyogenes *by polymerase-chain reaction (pcr) amplification. Specific restriction enzyme motifs for cloning were introduced into the amplified DNA fragment by using the oligonucleotide primer 5' CTCG CAA GAG GTA CAT ATG CAA CAA GAC 3' to produce a unique NdeI site, and 5' GCA GTA GGT AAG CTT GCC AAA AGC 3' to produce a unique HindIII site. The amplified DNA fragment was ligated into the EcoRI site of a pcr-cloning vector (Invitrogen) and the resulting plasmid was used to transform *E. coli *DH5α. The HindIII/EcoRI DNA fragment containing the full-length SpeA gene minus the signal peptide was cloned into pET24 vector for expression in *E. coli *BL21. Although proteins were also produced with the leader peptide sequence present, deletion of the leader peptide appeared to result in a higher yield of protein.

Two different mutants of SpeA were produced by changing amino acid residue leucine 42 to either arginine or alanine by using previously described methods [17]. The first SpeA construct consists of a single mutation at residue leucine 42 [SpeA (L42R) or SpeA (L42A)], while the second construct consists of a fusion between the SpeA (L42R) and a mutant SpeB protein. The wild-type SpeB zymogen, isolated from the same strain of *S. pyogenes *used to clone SpeA, was truncated by PCR cloning to produce the mature protein without the prosegment domain (noncatalytic). A mutant, catalytically-inactive SpeB [SpeB (C47S)] was constructed by site-specific mutagenesis of the DNA coding sequence, altering cysteine 47 to serine. This conservative change maintains the approximate dimensions of the active-site side chain but prevents proteolytic activity. The SpeB (C47S) DNA was used as a fusion partner with SpeA (L42R) that was constructed with the following oligonucleotide primers:

1. SpeA forward primer, including NdeI site:

5' GATATACATATGCAACAAGACCCCGATCCAAGCC 3'

2. SpeA reverse primer, with SpeB overlap:

5' GAGATTTAACAACTGGTTGCTTGGTTGTTAGGTAGAC 3'

3. SpeB forward primer, with SpeA overlap:

5' GTCTACCTAACAACCAAGCAACCAGTTGTTAAATCTC 3'

4. SpeB reverse primer; adding an Amber codon:

5' GAATTCGGATCCGCTAGCCTACAACAG 3'

For cloning, the SpeA (L42R) gene was used as a PCR template and primers 1 and 2 were used to prepare a double-stranded sequence overlapping with SpeB (C47S). A separate PCR reaction with the SpeB (C47S) gene insert using primers 3 and 4 was performed to generate a double-stranded DNA fragment overlapping with SpeA (L42R). The PCR fragments were purified by agarose gel electrophoresis and mixed together for a final PCR reaction using primers 1 and 4, to create the full-length gene fusion of SpeA (L42R)-SpeB (C47S). This full-length fragment was cloned into the vector pT7Blue (Novagen) and the sequence was confirmed.

### Protein production

The SpeA (L42R, L42A) and SpeA (L42R)-SpeB (C47S) fusion genes were subcloned into pET24b (+) for expression in *E. coli *BL21 host strains. Production of the recombinant proteins and purification methods were as previously described [17,18]. The endotoxin levels of protein preparations were less than detection limits, as determined by a limulus amebocyte lysate assay (Cambrex, Walkersville, MD). Purified wild-type SpeA and affinity-purified rabbit antibodies specific for either SpeA or SpeB were obtained from Toxin Technology (Sarasota, FL) and used to confirm identity of the recombinant proteins by Western blots. Proteins (2 μg/lane) were electrophoresed through 12% polyacrylamide gels in the presence of SDS (1%), with dithiothreitol (2 mM). Gels were then electroblotted onto a protein-binding membrane (Amersham), and blocked (2 h, 37°C) with 0.2% casein in PBS. The membrane was then incubated (1 h, 37°C) with a 1/200 dilution of affinity-purified, rabbit anti-SpeA or SpeB (Toxin Technologies, Sarasota, FL). Unbound antibody was washed from the membrane using PBS, and bound antibody was detected with peroxidase conjugated, goat anti-rabbit antisera, using a commercial color development kit (BioRad, Richmond, CA).

### HLA-DR/DQ binding assay

The DR1 homozygous, human B-lymphoblastoid cell line LG2 was used to detect binding of the SpeA proteins to MHC class II molecules, as previously described [17]. In brief, LG2 cells (4 × 10^5^/50 μl) were incubated 40 min (37°C) with wild-type or mutant SpeA in Hanks balanced salt solution (HBSS) containing 0.5% bovine serum albumin. The cells were washed with HBSS and then incubated with 5 μg of affinity-purified rabbit anti-SpeA antibody (Toxin Technology) for 1 h on ice. Unbound antibody was removed, and the cells were incubated with FITC-labeled goat anti-rabbit IgG (Organon Teknika Corp., Durham, NC) on ice for 30 min. The cells were washed and analyzed by flow cytometry (FACScan; Becton Dickinson & Co., Mountain View, CA). Controls consisted of cells incubated with affinity purified anti-TSST-1 and the FITC labeled antibody without prior addition of SpeA.

### T-lymphocyte responses

Lymphocyte proliferation was used to measure biological responses to the streptococcal proteins, as previously described [17]. Human peripheral blood mononuclear cells, obtained from consenting volunteers, were purified by Ficoll-hypaque (Sigma, St. Louis, MO) buoyant density gradient centrifugation. The cells were cultured in RPMI-1640 with 5% FBS for 72 h, and pulsed-labeled for 12 h with 1 μCi [^3^H]-thymidine (Amersham, Arlington Heights, IL). Cells were harvested onto glass fiber filters, and [^3^H]-thymidine incorporation into the cellular DNA was measured by a liquid scintillation counter (BetaPlate, Wallac Inc., Gaithersburg, MD).

### Serum antibody

Serum levels of total IgG were determined by enzyme-linked immunosorbent assays (ELISA). Polystyrene 96-well plates (Nunc) were coated with a 1 μg/ml solution of antigen in 0.05 M sodium carbonate buffer (pH 9.6) overnight (4°C). The plates were blocked for 2 h (37°C) with 0.2% casein in PBS (138 mM NaCl, 2.7 mM KCl) and then washed three times with PBS. Serum samples were serially diluted in 0.02% casein in PBS and incubated in the antigen-coated plates for 1 h (37°C). The plates were washed three times with PBS and a 1:2000 dilution of goat anti-mouse IgG, HRP conjugated (Southern Biotechnology), was added in 0.02% casein PBS. The plates were incubated for 60 min (37°C), washed three times with PBS and then developed (30 min, 22°C) with TMB substrate (3,3',5,5'-Tetramethylbenzidine, Pierce). The reactions were stopped with the addition of 0.5 M H_2_SO_4 _and the absorbance determined at 450 nm wavelength.

### Vaccinations

HLA-DQ8/human CD4+ transgenic mice were described previously [19]. Pathogen-free, 10–12-week-old BALB/c mice were obtained from Charles River (National Cancer Institute, Frederick, MD), maintained under pathogen-free conditions, and fed laboratory chow and water *ad libitum*. For vaccinations, mice were each injected 3 times (2 weeks between injections) intramuscularly (i.m.) with 10 μg of proteins (100 μl) combined with 100 μl MPL adjuvant (MPL™ + TDM+ CWS Emulsion, RIBI ImmunoChem Research, Inc., Hamilton, MT). This research was conducted in compliance with the Animal Welfare Act and other federal statutes and regulations relating to animals and experiments involving animals and adhered to the principles stated in the *Guide for the Care and Use of Laboratory Animals*, National Research Council, 1996.

### Bacterial sepsis and toxic shock

The β-hemolytic *Streptococcus pyogenes *strain RIID231 (*spea+, speb+*), a human laryngitis isolate, was used as a source of streptococcal genes and bacteria for mouse challenges. Bacteria were propagated in Todd-Hewitt broth cultures (0.2% yeast extract) and single colonies were isolated after growth on sheep blood agar plates containing the same media to prepare bacteria for challenge studies. Streptococci were collected from broth cultures in mid-log growth phase, washed three times by gentle centrifugation in PBS and the density (A_670_) was adjusted by using PBS (22°C). Colony-forming units were confirmed by growth of diluted bacteria on sheep blood agar plates. For mouse challenges, bacteria diluted in PBS were injected (10^5 ^CFU in 100 μL) into tail veins using a tuberculin needle and syringe (27 g), followed 4 h later by i.p. administration of 75 μg (50 μL) of *E. coli *lipopolysaccharide (LPS; Difco, Detroit, MI). For challenge with SpeA, mice were injected i.p. (50 μL) with toxin diluted in PBS.

## Results

### Vaccine design

**Figure 1:**
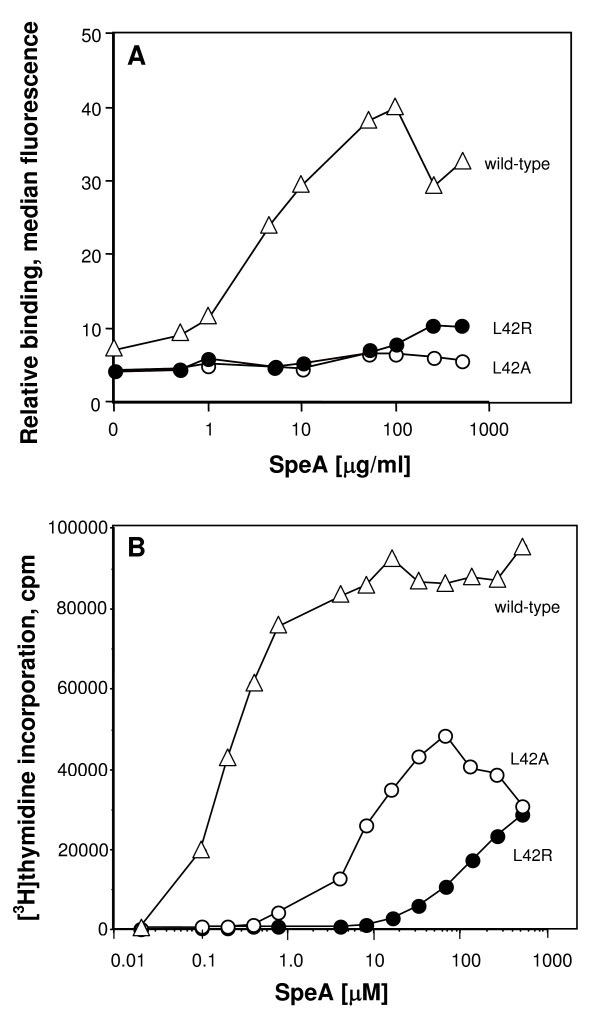
**Biological activity of SpeA mutants**. A. Mutations of amino acid position leucine 42 of SpeA to arginine or alanine resulted in greatly diminished interactions with cell surface MHC class II molecules, measured by laser fluorescence-activated flow cytometry and FITC-labeled rabbit anti-SpeA antibody. B. Mutations of amino acid position leucine 42 of SpeA to arginine or alanine resulted in greatly diminished activation of human lymphocytes. Human T-cell proliferation was assessed by [^3^H]thymidine incorporation (12 h pulse) after 60 h of culture. Each data point represents the mean of triplicate determinations; SEM ≤ 5%.

The genes encoding SpeA and SpeB were cloned from a strain of *S. pyogenes *originating from a patient with laryngitis. The binding interface between SpeA and human MHC class II molecules consists of contacts located in the N-terminal domain that are in common with other bacterial superantigens [17]. Leucine 42 of SpeA protrudes from a reverse turn on the surface of SpeA to potentially form a major hydrophobic contact with HLA-DQ or HLA-DR receptor molecules. Mutants of SpeA were constructed to alter leucine 42 (L42) and reduce HLA-DR binding. Mutations of the SpeA amino acid residue 42 to arginine or alanine (L42R or L42A) resulted in greatly diminished interactions with cell surface HLA-class II molecules (Figure 1A), as measured by flow cytometry.

Human T-cell proliferation in response to these mutants was next assessed. Both SpeA mutations of L42 resulted in greatly diminished activation of human lymphocytes (Figure 1B). Although alanine and arginine substitutions of L42 resulted in similar levels of attenuated MHC class II binding, arginine substitution (L42R) produced the greatest reduction of T-cell responses (Figure 1B) and was therefore chosen for further study.

**Figure 2:**
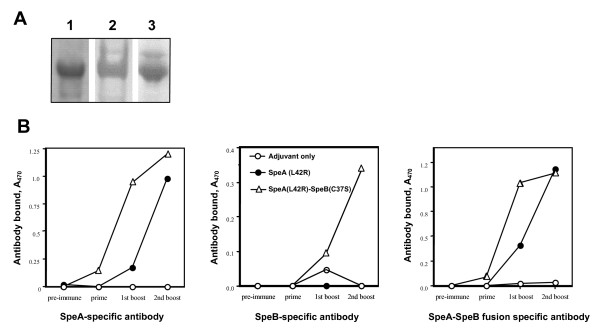
**Antibody recognition of SpeA (L42R)-SpeB (C47S) fusion protein**. A. Antibody recognition *in vitro*. Coomassie Blue stain of isolated SpeA (L42R)-SpeB (C47S), lane 1; Western blot using-affinity purified, rabbit anti-SpeB (lane 2) or anti-SpeA antibody (lane 3). B. Antibody response and recognition *in vivo*. Mice (BALB/c) were vaccinated three times with 10 μg of each protein and adjuvant (MPL), allowing two weeks between injections. Sera from each experimental group (n = 5) were pooled for measurement of specific antibodies. Data shown are antigen-specific antibodies (ELISA units) present in a 1:100,000 dilution of pooled sera from mice vaccinated with SpeA (L42R), SpeA (L42R)-SpeB (C47S) fusion or adjuvant only.

A catalytically inactive SpeB was constructed by mutating cysteine at position 47 [SpeB (C47S)] and used as a fusion partner with SpeA (L42R). The predicted 54 kDa protein was detected by polyacrylamide gel electrophoresis (Figure 2A). The SpeA (L42R)-SpeB (C47S) fusion was catalytically inactive towards peptide substrate (data not shown), using a previously reported assay [20]. In addition, rabbit antibodies specific for either SpeA or SpeB both detected SpeA (L42R)-SpeB (C47S) by Western blot analysis (Figure 2A). Although an additional recombinant protein was produced to incorporate the SpeB prosegment in the final SpeA-B fusion, this was not used further due to poor stability in solution.

### Mouse antibody response to SpeA (L42R)-SpeB (C47S) and protection from SpeA-toxic shock

**Figure 3:**
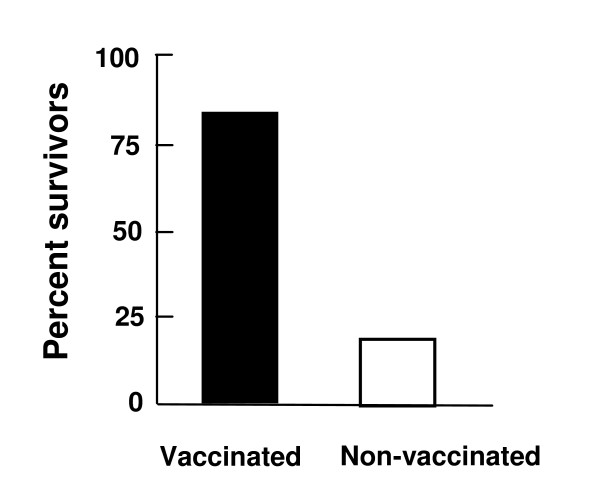
**Protection of transgenic HLA-DQ8 mice from *Streptococcus pyogenes *sepsis following vaccination with SpeA (L42R)-SpeB (C47S) fusion protein**. Mice (5 per group) were vaccinated three times with 10 μg of each protein with adjuvant (MPL), allowing two weeks between injections. Three weeks after the last vaccination the mice were injected (i.v) with 10 LD_50 _of *S. pyogenes *and survival was monitored for 10 days.

Immune recognition *in vivo *of the recombinant streptococcal proteins was next examined. BALB/c mice were vaccinated three times with 10 μg of SpeA (L42R) or SpeA (L42R)-SpeB (C47S), allowing two weeks between injections. Although vaccination with either SpeA (L42R) or the SpeA (L42R)-SpeB (C47S) produced high antibody titers, antibodies from SpeA (L42R) vaccination recognized only SpeA (Figure 2B), whereas, antibodies from the SpeA (L42R)-SpeB (C47S)-vaccinated mice recognized both SpeA and SpeB (Figure 2B). Seroconversion (IgG) occurred after the first vaccination with SpeA (L42R)-SpeB (C47S) compared to two injections required for the SpeA (L42R) vaccination (Figure 3). Although these data confirmed the potent immunogenicity of the SpeA constructs, the inbred mouse was an inadequate model to demonstrate protective immunity. Within reasonable physiological limits, wild-type SpeA was not lethal for several inbred mouse strains examined. Therefore, a transgenic model was used, consisting of C57BL/6 mice expressing human CD4 and HLA-DQ8 [21,22]. Wild-type SpeA was previously shown to be lethal at relatively low concentrations for the HLA-DQ8 mice [23]. The lymphocyte response from the HLA-DQ+ mice to SpeA (data not shown) was very similar in dose effect to those obtained with human mononuclear cells. Non-vaccinated HLA-DQ8 mice succumbed to SpeA challenge, whereas, vaccination with either SpeA (L42R) or SpeA (L42R)-SpeB (C47S) fully protected HLA-DQ8 transgenic mice from challenge with the same amount of wild-type SpeA (Table 1).

**Table 1:** Vaccination and Immune Protection: HLA-DQ8/human CD4 Transgenic Mice

Vaccination^1^	Challenge Survival^2^
SpeA (L42R)	100%
SpeA (L42R)-SpeB (C47S)	100%
Adjuvant only	0%

^1^Vaccinations at 0, 2 and 4 weeks (3 doses) with 10 μg of SpeA (L42R) or SpeA (L42R)-SpeB (C47S) in adjuvant or adjuvant only.

^2^Percent mice surviving wild-type SpeA challenge, 5 LD_50 _per mouse 2 weeks after last vaccination. 5 mice per group SpeA (L42R) and adjuvant only control; 4 mice for SpeA (L42R)-SpeB (C47S) vaccination. Experiments were performed twice with identical results.

### Vaccination with SpeA (L42R)-SpeB (C47S) and protection from streptococcal sepsis

Inconsistent results were obtained in attempts to model *S. pyogenes *sepsis in several inbred mouse strains. Therefore, the HLA-DQ8 transgenic mice were also used to examine vaccine efficacy in bacterial sepsis. Mice vaccinated as above were injected (i.v.) with live bacteria followed 4 h later by i.p. administration (75 μg) of *E. coli *LPS. Survival was monitored for 10 d after challenge. The co-administration of LPS, as previously documented for toxic shock [24], produced a measurable fatal disease (3–7 d) in mice injected with live *S. pyogenes *(Figure 3). The majority (80%) of vaccinated mice were protected from lethal sepsis in contrast to the unvaccinated control mice (Figure 3). Mice vaccinated with only SpeA (L42R) were not protected from bacterial sepsis (data not shown). However, it was unclear if these results were due to a limitation of the animal model or perhaps from the necessity to also target SpeB.

## Discussion

Because of the strong association between streptococcal toxic shock and invasive streptococcal infections [25], targeting SpeA is important for the development of a human vaccine for preventing or treating sepsis caused by *S. pyogenes*. The results presented indicated that a vaccine consisting of a fusion between the inactivated bacterial superantigen SpeA and the cysteinyl protease SpeB, formulated with an adjuvant, protected mice from lethal toxic shock syndrome induced by administration of biologically active SpeA. Further, vaccination with the SpeA (L42R)-SpeB (C47S) fusion protein protected HLA-DQ8 transgenic mice from lethal infection caused by a clinical isolate of *S. pyogenes*. The results from vaccinations of HLA-DQ8 transgenic mice demonstrated efficacy in a human-like, MHC class II receptor background, suggesting that SpeA (L42R)-SpeB (C47S) may be an important new vaccine for controlling streptococcal toxic shock and *S. pyogenes *infections. The potential advantages to this fusion protein above the isolated SpeA (L42R) are potent activation of immune responses, immune protection against a second virulence factor (SpeB), potential cost savings and simplification of vaccine production.

The rationale for selecting mutations only in the MHC-binding region of SpeA was based on previous results with staphylococcal superantigens demonstrating that the effect of mutations to the MHC binding site produced a greater attenuation of superantigen activity and lethality than mutations to the TCR-binding site [26] and that the residues involved in MHC class II binding are more conserved than those involved in binding to the TCR Vß chain [18,27]. The mode of protection stimulated by SpeA (L42R)-SpeB (C47S) was presumed to be antibody mediated, though this was not directly ascertained in the current study. It is possible that conformational changes in the protein structures of SpeA and SpeB due to production as a single polypeptide may impact vaccine efficacy by altering recognition of the native bacterial proteins. However, antibody recognition is likely to be maintained during vaccination with the fusion product because the native biological activities of SpeB and SpeA were eliminated by site-specific mutagenesis methods that cause minimal perturbation of protein structure [28,29].

Additional data corroborate SpeB and SpeA as rational targets for immune intervention. Both proteins were produced by M1 *S. pyogenes *during growth in human saliva, and growth was dependent on SpeB [30], suggesting a potential protective role for secretory antibodies. Further, it was reported that SpeB influences tissue tropism of *S. pyogenes *[31] and was proposed as a seromarker for infection equal in performance to standards currently in use [32]. The *S. pyogenes *serotype M1 that spread globally in the late 1980s and early 1990s harbored phage-borne SpeA [33] and another common superantigen SmeZ [34]. In addition, four of five recently re-emerging strains (emm49 genotype) isolated from severe invasive *S. pyogenes *patients in Japan were *speA*^+ ^[35] and a prophage remnant encoding SpeA was noted in a macrolide-resistant strain of serotype M6 *S. pyogenes *[36]. It was also noted that genes encoding SpeA are commonly found in pharyngeal *S. pyogenes *isolates [37].

Previous reports have suggested other surface proteins of *S. pyogenes *as potential candidates for vaccines [38,39], but strain variation is generally a complication. For example, though the M protein is universally expressed by *S. pyogenes*, antibody against one strain may not be protective against other strains due to varying susceptibility to opsonophagocytosis [40] resulting from differences in M protein structure. Vaccination with a fusion product of multiple M epitopes was reported as an alternative means to induce antibodies specific for dominant serotypes [39]. In contrast, mice actively immunized with SpeB resulted in non-type-specific immunity to challenge with heterologous *S. pyogenes*[41]. It is anticipated that vaccination with the SpeA-SpeB fusion protein described in the present study may result in protective immunity against multiple isolates of *S. pyogenes *due to the extensive antibody cross-reactivity previously observed among all sequence variants of SpeB [15] and the high frequency of SpeA-producing strains.

## Competing interests

The author declares that he has no competing interests.

## Authors' contributions

RGU conceived and performed the study. RGU wrote and approved the final manuscript draft.
